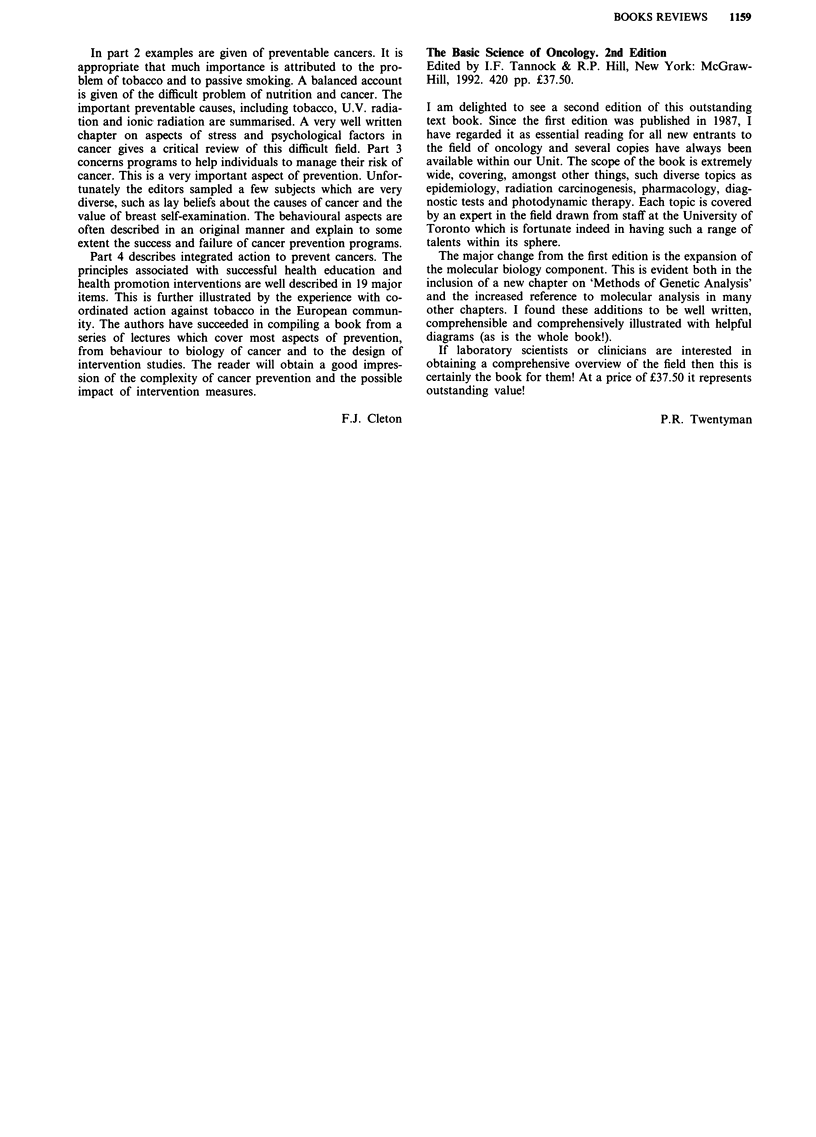# The Basic Science of Oncology. 2nd Edition

**Published:** 1993-05

**Authors:** P.R. Twentyman


**The Basic Science of Oncology. 2nd Edition**
Edited by I.F. Tannock & R.P. Hill, New York: McGraw-Hill, 1992. 420 pp. £37.50.

I am delighted to see a second edition of this outstanding text book. Since the first edition was published in 1987, I have regarded it as essential reading for all new entrants to the field of oncology and several copies have always been available within our Unit. The scope of the book is extremely wide, covering, amongst other things, such diverse topics as epidemiology, radiation carcinogenesis, pharmacology, diagnostic tests and photodynamic therapy. Each topic is covered by an expert in the field drawn from staff at the University of Toronto which is fortunate indeed in having such a range of talents within its sphere.

The major change from the first edition is the expansion of the molecular biology component. This is evident both in the inclusion of a new chapter on 'Methods of Genetic Analysis' and the increased reference to molecular analysis in many other chapters. I found these additions to be well written, comprehensible and comprehensively illustrated with helpful diagrams (as is the whole book!).

If laboratory scientists or clinicians are interested in obtaining a comprehensive overview of the field then this is certainly the book for them! At a price of £37.50 it represents outstanding value!

P.R. Twentyman